# MicroRNA-182 Alleviates Neuropathic Pain by Regulating Nav1.7 Following Spared Nerve Injury in Rats

**DOI:** 10.1038/s41598-018-34755-3

**Published:** 2018-11-13

**Authors:** Weihua Cai, Qingzan Zhao, Jinping Shao, Jingjing Zhang, Lei Li, Xiuhua Ren, Songxue Su, Qian Bai, Ming Li, Xuemei Chen, Jian Wang, Jing Cao, Weidong Zang

**Affiliations:** 1 Department of Human Anatomy, School of Basic Medical Sciences, Zhengzhou University, Henan, 450001 China; 2 Department of Anesthesiology, The Second Affiliated Hospital of Zhengzhou University, Henan, 450052 China; 3 Department of Anesthesiology and Critical Care Medicine, Johns Hopkins University School of Medicine, Baltimore, MD 21205 USA

## Abstract

The sodium channel 1.7 (Nav1.7), which is encoded by SCN9A gene, is involved in neuropathic pain. As crucial regulators of gene expression, many miRNAs have already gained importance in neuropathic pain, including miR-182, which is predicted to regulate the SCN9A gene. Nav1.7 expression in L4-L6 dorsal root ganglions (DRGs) can be up regulated by spared nerve injury (SNI), while miR-182 expression was down regulated following SNI model. Exploring the connection between Nav1.7 and miR-182 may facilitate the development of a better-targeted therapy. In the current study, direct pairing of miR-182 with the SCN9A gene was verified using a luciferase assay *in vitro*. Over-expression of miR-182 via microinjection of miR-182 agomir reversed the abnormal increase of Nav1.7 at both mRNA and protein level in L4-6 DRGs of SNI rats, and significantly attenuated the hypersensitivity to mechanical stimulus in the rats. In contrast, administration of miR-182 antagomir enhanced the Nav1.7 expression at both mRNA and protein level in L4-6 DRGs, companied with the generation of mechanical hypersensitivity in naïve rats. Collectively, we concluded that miR-182 can alleviate SNI- induced neuropathic pain through regulating Nav1.7 in rats.

## Introduction

Neuropathic pain has been widely recognized as “pain arising as a direct consequence of a lesion or disease affecting the somatosensory system,” since the International Association for the Study of Pain redefinition in 2008. Patients who experience neuropathic pain often live lower quality lives due to this disorder. However, the treatment of neuropathic pain has been encumbered by the poorly understood molecular mechanisms. Peripheral nerve injury is one of the causes of neuropathic pain. Following nerve injury, ectopic hyperactivity of afferent neurons occurs in the DRGs due to the increased expression of voltage-gated sodium channels (Navs), mediating various enduring changes in the nervous system^1–5^. Navs contain a large pore-forming α subunit and auxiliary β subunits^6^. The genes for Nav1.1 to Nav1.9 encode for distinct channel isoforms (SCN1A to SCN5A, SCN8A, SCN9A, SCN10A, and SCN11A), each displaying specific properties^7^. Clear evidence showed that Navs (e.g. Nav1.3, Nav1.7, Nav1.8, and Nav1.9) involved in the initiation and generation of action potentials in neurons in both the central and peripheral nervous systems, are important components in pain pathways^8–10^. An increase in Navs after nerve injury was reported to result in ectopic spontaneous activity of afferent neurons^10,11^.

Among Navs, the Nav1.7 whose α subunit is encoded by SCN9A, is specifically expressed in primary somatosensory neurons, which are afferent neurons specialized for nociception^12^. Nav1.7 mainly accumulates at fiber endings and amplifies small subthreshold depolarizations, suggesting it to be a threshold channel that regulates neuron excitability^13^. However, previous studies on the role of Nav1.7 in pain seem to have inconsistent results. In humans, Nav1.7 mutation is reported to result in either congenital insensitivity or severe episodic hypersensitivity to pain^14–20^. Moreover, as voltage-gated sodium channels can be pharmacologically classified as tetrodotoxin-sensitive and tetrodotoxin-resistant, Nav1.7 is one of tetrodotoxin-sensitive sodium channels. The expression levels or tetrodotoxin-sensitive current density are increased in the DRG neurons in inflammatory pain, diabetic neuropathic pain, burn pain, and phantom limb pain^13,21–23^. Though some studies suggest that Nav1.7 expression is down regulated in SNI injured DRGs in mice and rats^24,25^, a significant increase in Nav1.7 current after SNI in rats was observed^7^. This may due to the SNI model, where severed and intact nerves are intermingled in the same DRG, inducing cross-excitation between cell bodies as well as fibers^7^. Our previous study also showed that Nav1.7 protein and mRNA expression was strongly increased in relative DRGs of chronic constriction injury-induced chronic pain in rats^26^. What’s more, our preliminary experiment suggested that, regardless of declined expression in spinal nerve ligation-related DRG neurons, Nav1.7 significantly increased in the DRG neurons from chronic constriction injury and in SNI rats. Thus, in this current study, we focused on the mechanism of Nav1.7 in SNI-induced neuropathic pain to tap into its potential for neuropathic pain therapy/analgesic therapy development.

MicroRNAs (miRNAs) are a class of non-coding RNAs that regulate mRNA translation repression and/or transcript degradation^27,28^. MiRNAs play essential roles in different neuropathic pain models, such as chronic constriction injury^29,30^, spinal cord injury^31^, spinal nerve ligation^32^, and diabetic neuropathy rat models^33^. Recently, several studies have suggested that miRNAs may alter gene regulation and protein expression as a consequence of neuropathic pain^5,34,35^.

In preliminary experiments, miR-31-5p, miR-22, miR-182, and miR-30b were found to be deregulated following SNI. It was predicted that Nav1.7 is one of the miR-182 target genes in rats by Target Scan (Target Scan Human, release 6.2). Until now, there were no confirmed studies on the regulation of Nav1.7 expression by miR-182 after SNI. In the current study, we investigated whether miR-182 can critically regulate neuropathic pain behavior, and whether this effect is mediated by Nav1.7 in the DRGs of SNI rats.

## Results

### SCN9A expression is increased in L4-6 DRGs neurons following spared nerve injury and this contributes to neuropathic pain

**Figure 1:**
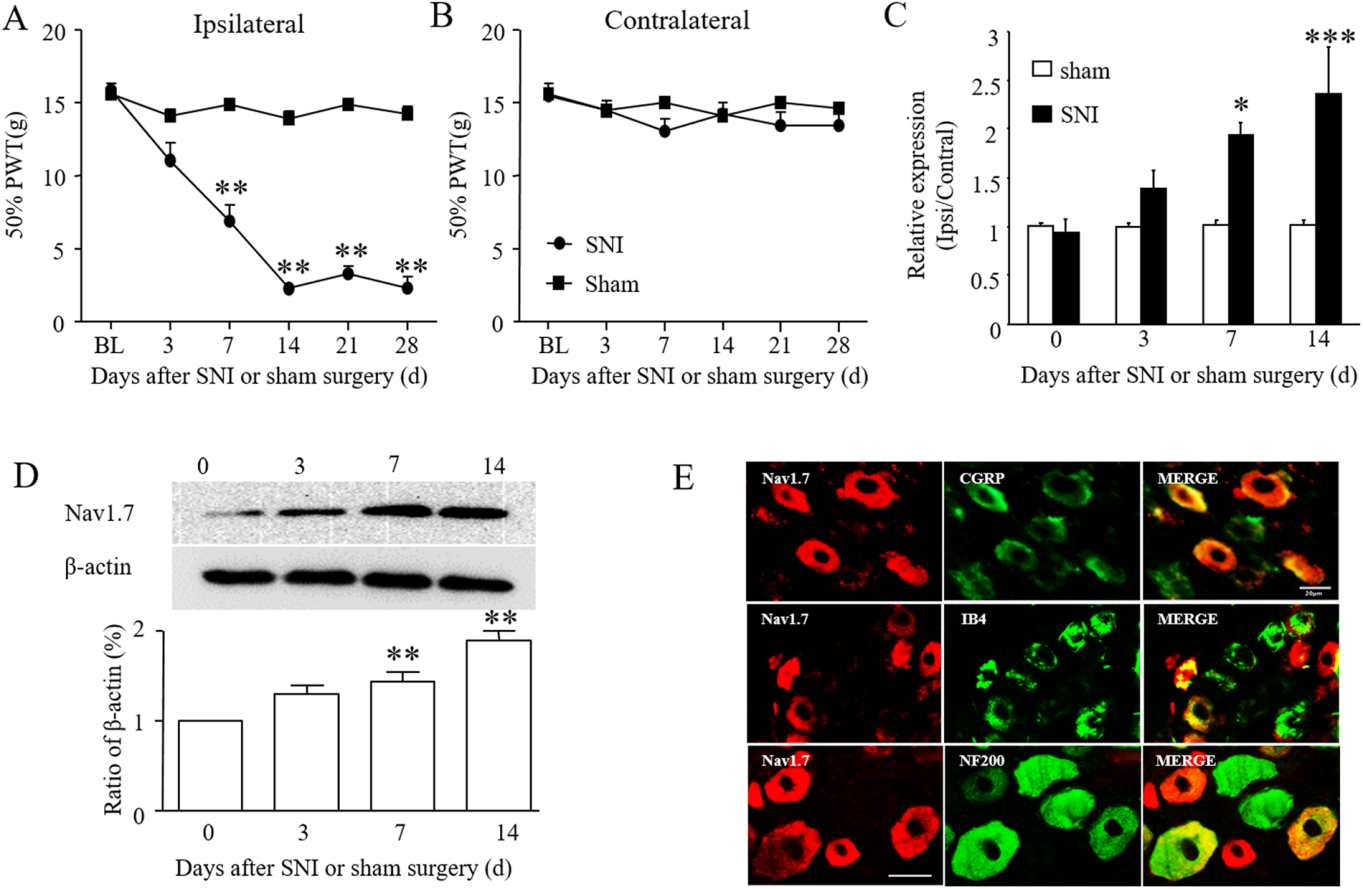
Spared nerve injury-induced mechanical hypersensitivity and Nav1.7 activation in rat injured DRGs. (**A**,**B**) Ipsilateral (**A**) and contralateral (**B**) paw withdrawal threshold (PWL) before or after SNI or sham surgery. **P < 0.01 vs. the baseline (BL). n = 6 rats/group. Two-way ANOVA followed by post hoc Tukey, *F*_1,5_ = 22.63 in (**A**), *F*_1,5_ = 1.809 in (**B**). (**C**) Levels of SCN9A mRNA in L4-6 DRGs at days shown after SNI or sham surgery in rats. n = 3 rats per group per time point. One-way ANOVA followed by post hoc Tukey, *F*_3,8_ = 51.67 for SNI group, *F*_3,8_ = 2.026 for sham group. (**D**) Levels of Nav1.7 protein in L4-6 DRGs at days shown after SNI surgery in rats. One-way ANOVA followed by post hoc Tukey, *F*_3,8_ = 0.9785 in contralateral side (Cont), *F*_3,8_ = 109.7 in ipsilateral side (Ipsi) *P < 0.05, **P < 0.01, ***P < 0.001 vs. the zero day. n = 3 for each time point. (**E**) Co-localization of Nav1.7 with CGRP, IB4, and NF200 in rat L5 DRG, respectively. Scale bar: 100 μm.

Spared nerve injury produced mechanical hypersensitivity, evidenced by a reduction in paw withdrawal threshold and hypersensitivity developed only on the ipsilateral side, and not the contralateral side. Mechanical hypersensitivity presented 3 days after surgery, and then reached a maximum 14 days after surgery, and lasted for at least 28 days (Fig. 1A,B). Time-dependent Nav1.7 expression in the ipsilateral L4-6 DRGs after SNI or sham surgery was then assessed. Compared with sham group rats, SCN9A expression increased from 3 days after surgery, and maintained high expression at 14 days after surgery in SNI group rats as compared to the sham surgery group (Fig. 1C). The western blot results further confirmed the significant increase in Nav1.7 protein level in the ipsilateral L4-6 DRGs at 14 days after SNI surgery (Fig. 1D; *p* < 0.001, one-way ANOVA). This data suggests that SCN9A in DRGs may have an important role in neuropathic pain.

To define the cellular localization of Nav1.7 in the DRGs, immunofluorescence double-labeling was performed. The results showed that the Nav1.7-positive signal co-located with CGRP, IB4, NF200, and was mostly on neurons that were co-stained for CGRP and IB4 (Fig. 1E).

### miR-182 targets SCN9A in DRG neurons

**Figure 2:**
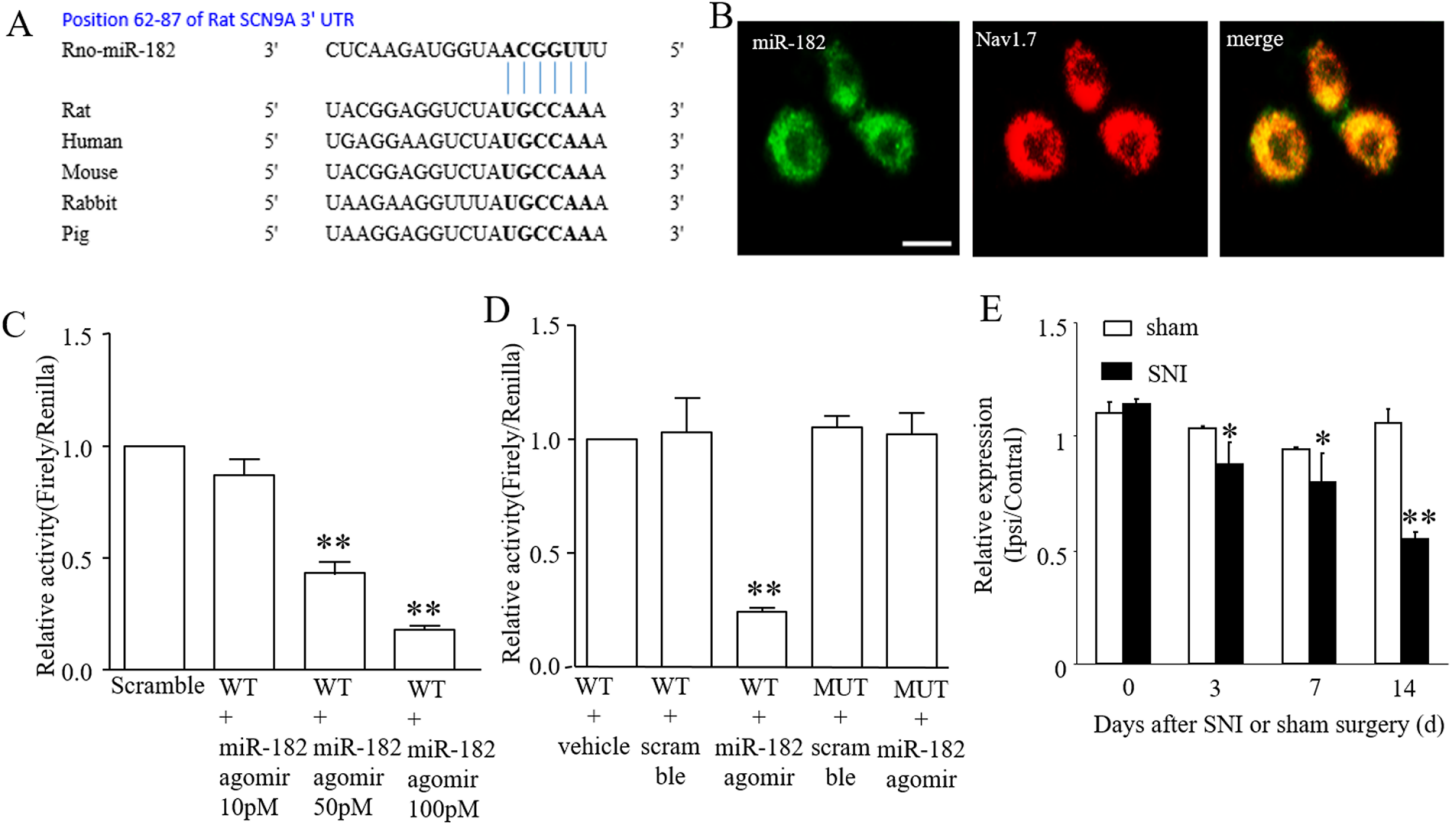
miR-182 directly targets SCN9A 3′UTR. (**A**) The binding site of miR-182 within SCN9A 3′-UTR. The seed sequence is stressed in bold. (**B**) Co-localization of miR-182 with Nav1.7 in rat L5 DRG. Scale bar: 100 μm. (**C**) miR-182 agomir dose-dependently decreased the relative activity in PC12 cells transfected with SCN9A3′-UTR. One-way ANOVA followed by Tukey’s Multiple Comparison Test. *F*_3,8_ = 86.367, **P < 0.01 vs. NC. n = 3 for each treatment. (**D**) The relative activity of luciferase in PC12 cells with wild type and mutant SCN9A 3′UTR after transfected with miR-182 agomir or scramble. n = 3 for each treatment. *F*_4,10_ = 37.508. **P < 0.01 vs. WT+ vehicle. (**E**) Levels of miR-182 in L4-6 DRGs at days shown after SNI or sham surgery in rats. *F*_3,8_ = 74.885 in SNI group, *F*_3,8_ = 0.123 in sham group. **P < 0.01 vs. the baseline. n = 6 for each treatment.

miRNAs are known to inhibit the translation of specific genes by binding to their messenger RNA 3′UTR sequences. The sequence alignment of miR-182 and its binding site in the 3′UTR of SCN9A, is highly conserved among mammals (Fig. 2A). The expressed correlation of miR-182 with that of Nav1.7 was confirmed using ISH combined with immunofluorescence. The result showed that the Nav1.7 protein was completely co-localized with miR-182 in DRG neurons (Fig. 2B). The luciferase reporter assay results suggested that, in SCN9A 3′UTR vector-transfected PC12 cells, the renilla/firefly value of luciferase was dose-dependently decreased by transfection with miR-182 agomir (Fig. 2B), with a significant decrease from 10 pM to 100 pM miR-182 agomir and a 75.74% decrease occurred at 50 pM miR-182 agomir group when compared with the NC group. This indicates the presence of a miR-182 target site in the SCN9A 3′UTR. However, the renilla/firefly value of luciferase activity was not affected in the SCN9A mutation group (*P* > 0.05, one-way ANOVA, Fig. 2C,D).

To check whether miR-182 is related to neuropathic pain, the expression of miR-182 in the L4-6 DRGs of SNI group and sham group rats was assessed. The real-time PCR results indicated that miR-182 expression was decreased in the ipsilateral DRGs of SNI axotomy. As shown in Fig. 2E, the expression of miR-182 gradually declined from 3 days to at least 14 days after surgery in the SNI rats (*P < 0.01, one-way ANOVA), while the expression of miR-182 did not change in the sham-treated animals (*P* > 0.05, one-way ANOVA).

### MiR-182 regulates SCN9A expression in DRG neurons

**Figure 3:**
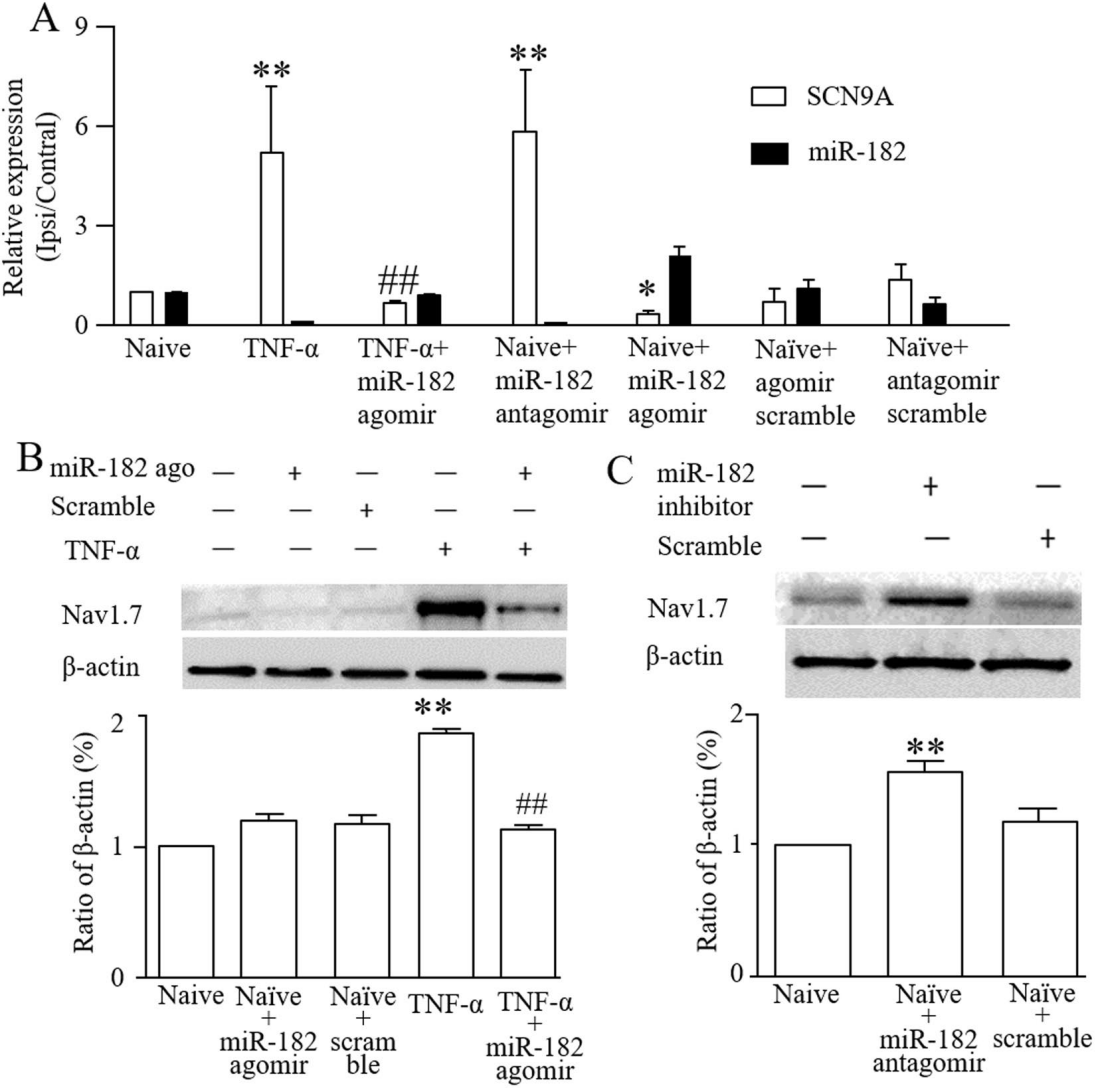
Administration of miR-182 affects Nav1.7 mRNA and protein levels in primary cultured DRG cells. (**A**) SCN9A mRNA and miR-182 expression in normal and TNF-α stimulated DRG cells after transfected with miR-182 agomir, miR-182 antagomir or scramble. (**B**) Level of Nav1.7 protein in normal and TNF-α stimulated DRG cells after transfected with miR-182 agomir or scramble. One-way ANOVA followed by *post hoc* Tukey, *F*_4,10_ = 65.06 (**C**) Level of Nav1.7 protein increased after transfected with miR-182 antagomir. One-way ANOVA followed by *post hoc* Tukey, *F*_2,6_ = 71.86, *P < 0.05, **P < 0.01 *vs*. the naive group; ^##^*P* < 0.01 *vs*. the TNF-α group. *n* = 3 for each treatment.

To investigate the effect of miR-182 on SCN9A expression, miR-182 agomir or antagomir was transfected into DRG cells. TNF-α treatment caused the up-regulation of SCN9A expression, and miR-182 agomir transfection directly ameliorated the abnormal SCN9A expression, concurrent with increased miR-182 expression. In contrast, transfection with a miR-182 antagomir decreased miR-182 expression, while increasing SCN9A expression in the DRG cells (Fig. 3A). The western blot results showed that miR-182 agomir transfection decreased the over-expression of Nav1.7 protein stimulated by TNF-α (Fig. 3B), while down-regulating miR-182 expression through transfection with a miR-182 antagomir increased Nav1.7 expression (Fig. 3C).

### Microinjection of a miR-182 agomir alleviates mechanical allodynia in SNI rats

**Figure 4:**
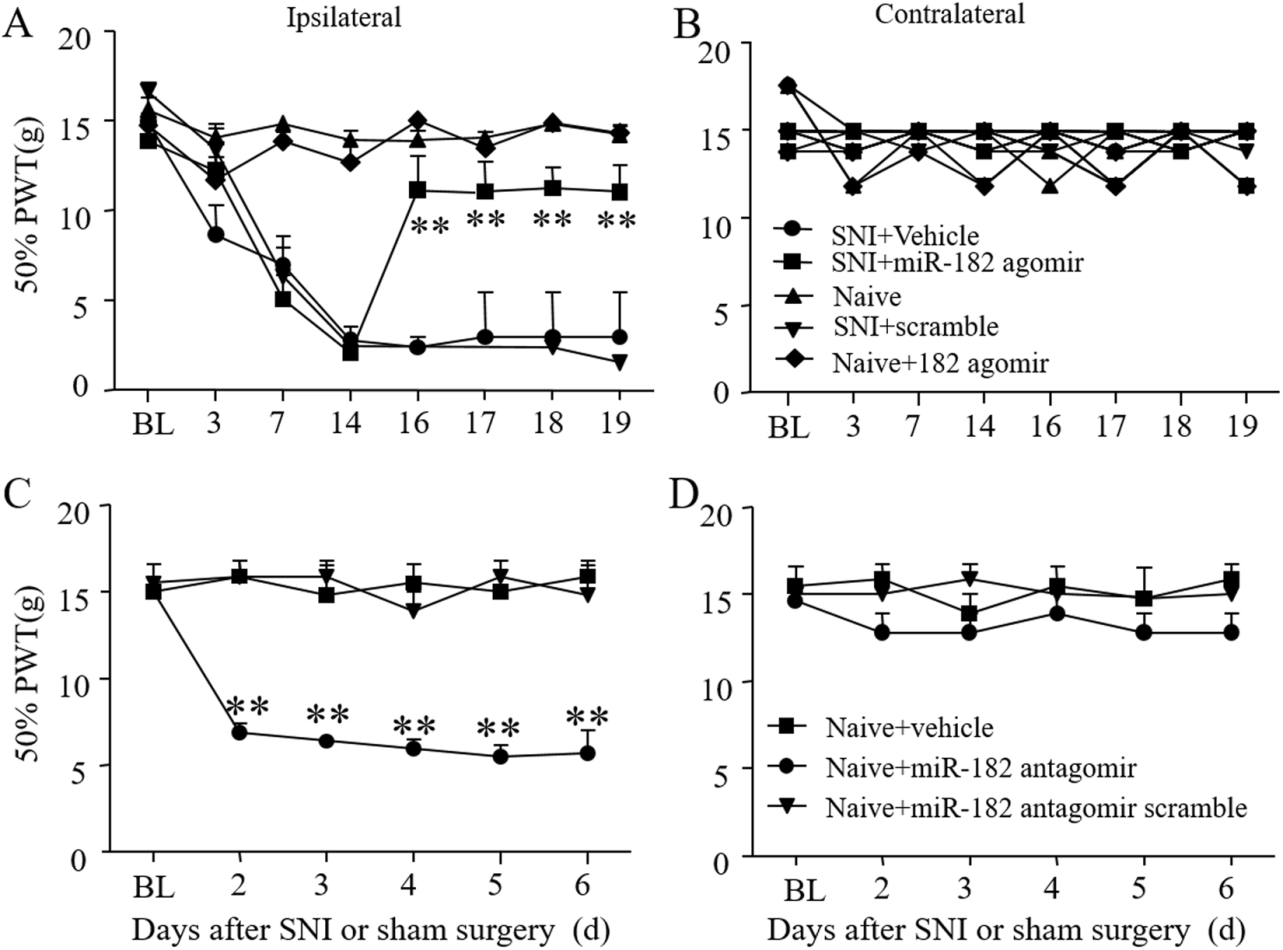
DRG microinjection of miR-182 agomir attenuates mechanical hypersensitivity in spared nerve injury rats. (**A**) Treatment with miR-182 agomir significantly attenuated the SNI-induced mechanical pain on ipsilateral side. (**B**) And with no effect on contralateral side. Two-way ANOVA followed by *post hoc* Tukey, *F*_1,8_ = 11.70 in ipsilateral side, *F*_1,8_ = 2.658 in contralateral side (**C**) miR-182 antagomir induced pain in normal rat on ipsilateral side. (**D**) And with no effect on contralateral side. Two-way ANOVA followed by *post hoc* Tukey, *F*_3,5_ = 14.26 in ipsilateral side, *F*_3,5_ = 0.3517 in contralateral side, **P < 0.01 *vs*. the baseline. *n* = 6 for each treatment.

The paw withdrawal threshold was measured to evaluate the pain threshold of the SNI rats. Pain was apparent in the ipsilateral side of the SNI rats starting from 3 days to at least 28 days after surgery, while the contralateral side demonstrated no change. Then, we tried to *in vivo* deliver miR-182 agomir/antagomir through DRG microinjection to rats. At 16 hours after injecting a negative control sequence marked by a 5′-FAM, fluorescence was detected in both DRG neurons and glia, suggesting the injected sequence is actually taken up by DRG cells (Fig. S2). Moreover, we found that miR-182 agomir relieved the pain condition, whereby the ipsilateral paw withdrawal threshold was significantly increased it (Fig. 4A) while no change was observed on contralateral side (Fig. 4B). In contrast, administration of a miR-182 antagomir induced mechanical pain, leading to a decrease in the ipsilateral paw withdrawal threshold in naïve rats (Fig. 4C,D).

### DRG microinjection of a miR-182 agomir inhibits the expression of SCN9A in L4-6 DRGs in SNI rats

**Figure 5:**
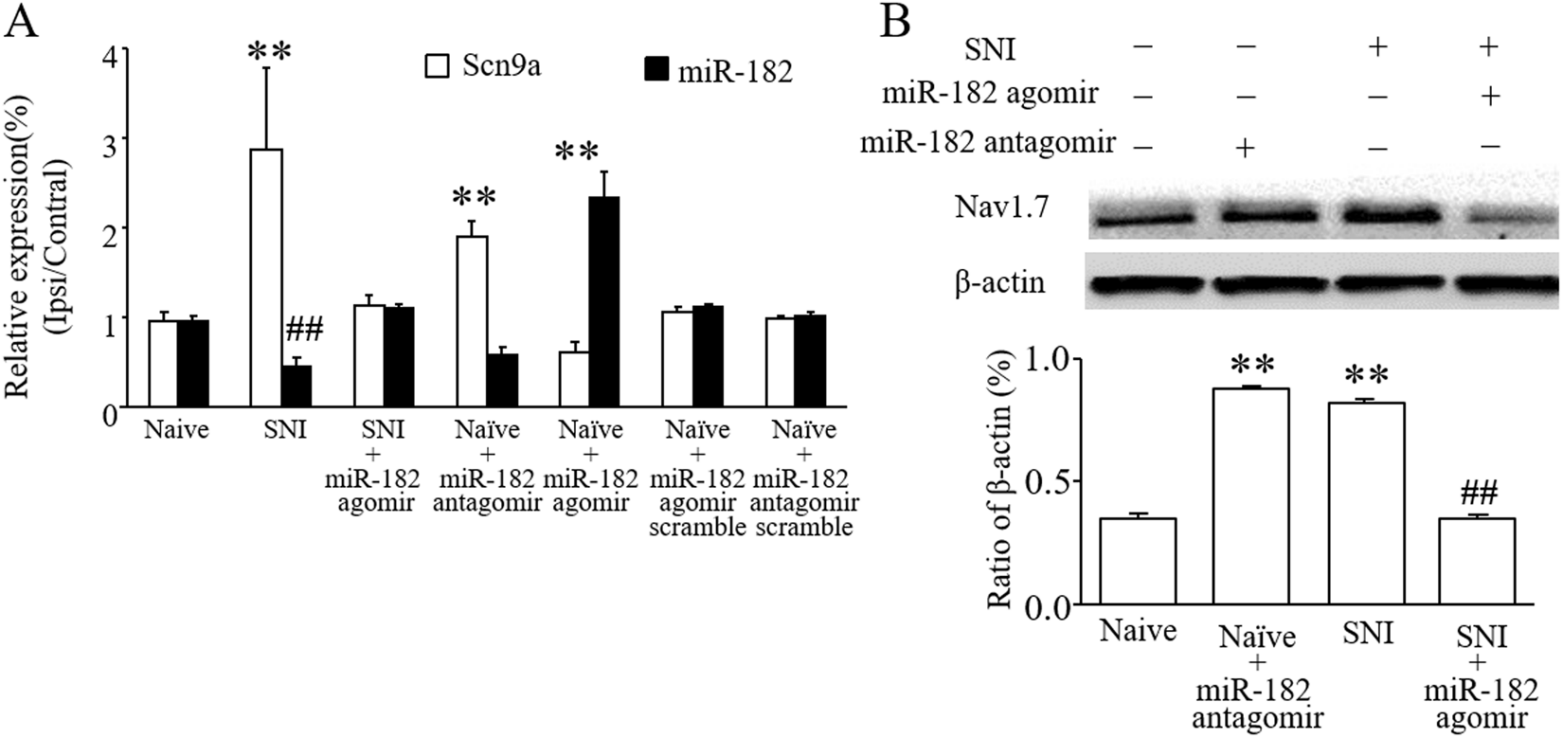
DRG microinjection of miR-182 agomir relieved abnormal increased Nav1.7 mRNA and protein level in spared nerve injury rats. (**A**) Administration with miR-182 agomir alleviated abnormal SCN9A mRNA level. One-way ANOVA followed by *post hoc* Tukey, *F*_6,14_ = 380.73 for SCN9A expression, *F*_6,14_ = 78.917 for miRNA-182 expression (**B**) as well as Nav1.7 protein expression in L4-6 DRGs of spare nerve injury rats. One-way ANOVA followed by *post hoc* Tukey, *F*_3,8_ = 57.584, **P < 0.01 *vs*. naive group; ^##^*P* < 0.01 *vs*. the SNI group. *n* = 3 for each treatment.

In order to further characterize the relationship between miR-182 and Nav1.7 in DRGs after SNI, L4-6 DRGs were isolated and mRNA and proteins levels were measured using real-time PCR and western blotting, respectively. Nav1.7 mRNA level was reduced after administration with miR-182 agomir in SNI-group rats, concurrent with increased miR-182 expression. In contrast, inhibiting miR-182 expression enhanced SCN9A expression (Fig. 5A). Meanwhile, Nav1.7 protein expression was reduced to normal levels after DRG microinjection of miR-182 agomir in SNI rats or increased, even in naïve rats, when miR-182 was inhibited (Fig. 5B).

### MiR-182 agomir treatment reduces SNI-induced abnormal excitability of small DRG neurons

**Figure 6:**
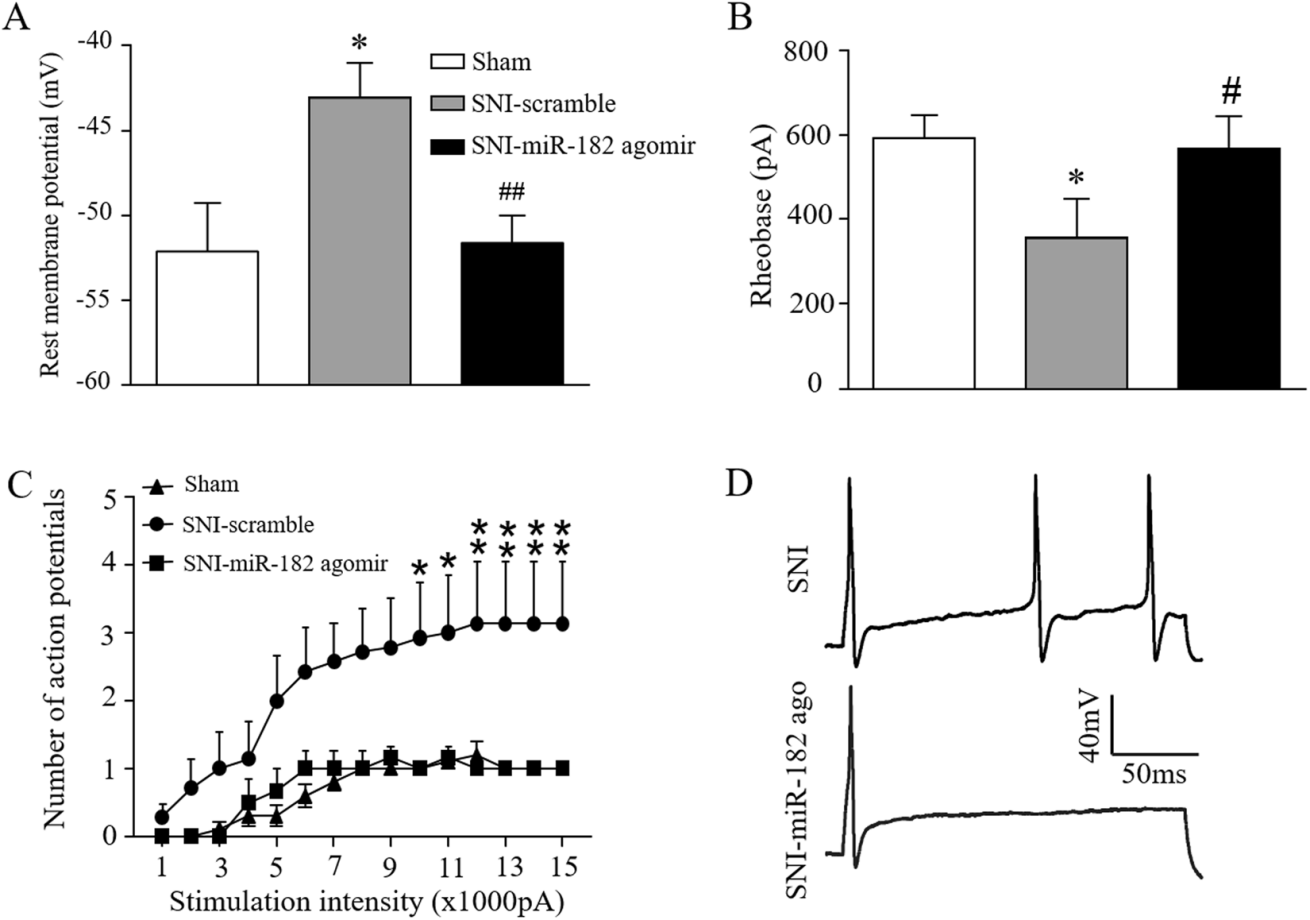
miR-182 treatment reduces SNI-induced abnormal excitability in L4 and L5 DRG small neurons of rats. (**A**) Resting membrane potential of small DRG neurons before SNI or after 7 days SNI with or without miR-182 agomir treatment. *P < 0.05 *vs*. sham and ^##^*P* < 0.01 *vs*. SNI-scramble by two-tailed unpaired Student’s *t*-test. (**B**) Rheobase for action potentials of small DRG neurons before SNI or after 7 days SNI with or without miR-182 agomir treatment. *n* = 10, 7 and 6 neurons from Sham (5 rats), SNI-Scramble (5 rats), and SNI-miR182 groups (3 rats). *P < 0.05 *vs*. sham and ^#^*P* < 0.05 *vs*. SNI-scramble by two-tailed unpaired Student’s *t*-test. (**C**) Numbers of evoked action potentials from the Sham, SNI-Scramble and SNI-miR-182 group after application of different currents. Two-way ANOVA followed by *post hoc* Tukey test, *F*_group_ (2, 20) = 76.24, *P < 0.05, **P < 0.01 *vs*. the same stimulation intensity in the SNI-miR-182 group. (**D**) Representative traces of evoked action potentials.

Since Navs play a key role in neuron excitability, we finally investigated whether neuronal excitability of small DRG neurons was affected by SNI using whole-cell current-clamp recording. We found that, RMP of small DRG neurons showed a significant increase after SNI surgery (*p* < 0.05, two-tailed unpaired Student’s *t*-test), which was restored by miR-182 agomir treatment (*p* < 0.01, two-tailed unpaired Student’s *t*-test) (Fig. 6A). The rheobase of small DRG neurons was significantly decreased by SNI (Fig. 6B). Although not significant, the rheobase was remedied through injection of miR-182 agomir. Moreover, the numbers of APs evoked by stimulation of ≥1000 pA was significantly decreased in miR-182 agomir injection group when compared to those in SNI group (Two-way ANOVA followed by *post hoc* Tukey test). The data of RMP, rheobase, and AP numbers indicate the remission of SNI-induced abnormal excitability of small DRG neurons by miR-182 treatment.

## Discussion

In the current study, the miR-182 regulation of Nav1.7 to participate in neuropathic pain was investigated at the RNA, protein, cellular, and behavioral levels. Our results indicated that miR-182 decrease following SNI enhanced the expression of Nav1.7, resulting in mechanical hypersensitivity. Rescuing the fall of miR-182 through microinjection of miR-182 agomir relived SNI-induced hyperalgesia.

The results also confirmed that Nav1.7 protein and mRNA expression was significantly increased in the L4-6 DRGs of SNI rats, which is consistent with our previous study^36^. Currently, many researchers focus on blocking sodium channels to treat neuropathic pain^37,38^. Specific and direct knockdown of Nav1.7 using a herpes vector has been shown to alleviate inflammatory hyperalgesia^39^. However, it is still not possible to specifically inhibit Nav1.7 in NP without side effects.

Considering the fact that epigenetic modification methods such as DNA methylation, histone acetylation, non-coding RNAs and microRNA mimics were widely used to regulate gene expression in recent years, we hypothesized that miRNAs may help us reach such a goal. In preliminary study, miR-30b^10^, miR-182 (current study), miR-103 (unpublished data) *et al*. was reduced in neuropathic pain model rats. In current study, we regulated the Nav1.7 expression using miR-182 agomir and antagomir with hardly any physical or psychological side effects. More importantly, mechanical hypersensitivity was distinctly relieved as a result of miR-182 agomir injection, verifying the effectiveness of miR-182 in neuropathic pain treatment. In fact, several publications dealing with miRNAs and neuropathic pain have revealed that miRNAs would provide new insights into pain generation and processing as well as relieving^40,41^.

However, it should be noted that DRG SCN9A mRNA down-regulation and Nav1.7 protein increase following peripheral nerve injury might be the result of various epigenetic changes or other possible mechanisms. Whether these mechanisms exist and how they work together are, however, unclear and call for future studies. Thus, plenty of additional investigations are needed before the precise and high-efficient application of miRNAs as therapies.

Moreover, the adequate choice of time point for administration of a therapy can be considered as a crucial issue, in not only experimental design but also in clinical therapy. In the current study, the DRG microinjection of miR-182 agomir/antagomir was performed on 14 days after surgery when the neuropathic pain was stable. As the current study mainly focuses on expression changes in DRG neurons, DRG microinjection was used^42^. Although this is an efficient method of administration, it may be difficult for clinical application. Moreover, the duration of efficacy following a direct injection of miRNA is short. Using viral vectors and replacing the DRG microinjection with an intrathecal administration method may have better clinical value.

Ectopic firing and hyperexcitability that occur in DRG neurons induced by nerve injury and the following long-term changes such as periphery sensitization are considered to be a key role in the genesis of neuropathic pain^43,44^. Nerve injury-induced high RMP and low rheobase in the small-diameter DRG neurons were found in the relative DRG neurons following SNI surgery. Meanwhile, our result showed that Nav1.7 was preferentially expressed in small-diameter DRG neurons. Given the knowledge that neuron RMP is mainly maintained by Na + potentials^45^, we assume at least part of the change in neuron excitability to be the presentation of increased quantity or enhanced function of Nav1.7. This result suggested that Nav1.7 may contribute to neuropathic pain development through changing relative neuron excitability. More importantly, those changes might be rescued by miR-182 agomir treatment, reconfirming the regulating mechanism of miR-182 to Nav1.7 in neuropathic pain. However, aside from identifying Nav1.7 as a target of miR-182, future studies are needed to investigate the mechanisms underlying this pathway and the role and targets of additional miRNAs in maintaining and relieving neuropathic pain.

In conclusion, the evidence presented here suggests that increased expression of SCN9A following SNI resulted in Nav1.7 up-regulation, enhanced excitability of DRG neurons, and contributed to neuropathic pain and that miR-182 might interact with this SCN9A gene to reduce Nav1.7 expression and alleviated mechanical hypersensitivity.

## Methods

### Animals

Male Sprague-Dawley rats weighing 200–300 g were obtained from the Animal Experiment Center of Henan Province. The rats were housed in a facility that was kept in a standard 12-hour light/dark cycle, with available access to water and food. Animals were housed for 1–2 d before behavioral testing commenced. Handling and care of all animals were approved by and adhere to ethical guidelines set by the Life Science Ethics Committee of Zhengzhou University. All methods were performed according to the relevant guidelines and regulations of the International Association for the Study of Pain.

### Behavioral tests

Mechanical behavioral tests were carried out as described previously^46^. Paw withdrawal thresholds to mechanical stimuli were measured using the up–down test method, as described previously^47^. Briefly, rats were placed in a transparent box on an elevated mesh screen. Von Frey filaments, ordered in force log increments (0.4,0.6,1.0,2.0,4.0,6.0,8.0,15.0 g) were applied to the left and right hind paws, on the plantar surface. The 2.0 g stimulus was used first. If a positive response occurred, the smaller von Frey hair (1.0 g) was used; if a negative response was observed, then the next larger hair would be used. The test was performed when (i) a negative response was obtained using the 15.0 g stimuli or (ii) three positive responses followed the first stimuli. The paw withdrawal threshold was determined by transforming the model of positive and negative responses into a 50% threshold value on the von Frey filament stimulation using a formula provided by Dixon^11^.

### Spared nerve injury model

The SNI model was implemented in Sprague-Dawley rats following established procedures^48^. Briefly, rats were anesthetized using isoflurane, and the tibial nerve and common peroneal nerve were isolated and then ligated using a 3–0 silk suture. The rest of the peripheral branch of the sciatic nerve and the sural nerve remained intact without any contact or stretching. Finally, the incision was closed.

### Drug administration and DRG microinjection

The miR-182 agomir (5′-UUUGGCAAUGGUAGAACYCACACCG-3′), its scrambled negative control (5′-UUC UCC GAA CGU GUC ACG UTT-3′), the miR-182 antagomir (5′-CGG UGU GAG UUC UAC CAU UGC CAA A-3′), and its scrambled negative control (5′-CAG UAC UUU UGU GUA GUA CAA-3′) were synthesized by GenePharma (Shanghai, China). Before the DRG microinjection, they were mixed with invivofectamine® 3.0 transfection reagent (Invitrogen, Carlsbad, CA).

The DRG microinjection was performed as previously described^49^. In brief, a midline incision was made in the lower lumbar back region to expose the unilateral L4 and L5 DRG. The mixed miR-182 agomir/antagomir (20 µM, 2 µL) was injected into the DRG using a glass micropipette (tip diameter 20–40 µm) which was connected to a Hamilton syringe^50,51^. The same volume of saline was used as a control. The micropipette was removed 10 min after administration. The surgical field was washed using sterile saline and the incision was closed.

### Primary DRG neuron culture and transfection

Primary DRG neuron culture and transfection were performed as previously described^49^. In brief, rats (3 weeks old) were euthanized using isoflurane and then their DRGs were collected in cold Neurobasal Medium (Thermo Fisher Scientific) with 10% fetal bovine serum (FBS; Gibco), 100 µg/mL streptomycin, and 100 units/mL penicillin (Gibco). The DRGs were then treated using a mixed enzyme solution (1 mg/mL collagenase I, 5 mg/mL dispase II in Neurobasal Medium). Dissociated cells were collected in mixed Neurobasal Medium and plated in a six-well plate pre-coated with poly-L-lysine (Sigma). The cells were cultured in 5% CO_2_ at 37 °C. After 1 day, 5 µL of miR-182 agomir/antagomir or negative control (20 µM; GenePharma, Shanghai, China) was diluted using 100 µL of Neurobasal Medium for 5 min. Meanwhile, 1 µL of lipo2000 (Invitrogen, Carlsbad, CA) was diluted using 100 µL of Neurobasal Medium for 5 min, and then the two were mixed for 25 min. Finally, the mixture was placed into each 2 mL well and an additional 800 µL of Neurobasal Medium was added. The cells were collected 30 h later. The cells were treated in the fresh medium without or with 100 ng/mL tumor necrosis factor-α (TNF-α) for 30 minutes after 3 d of culture^52^.

### Luciferase assay

The sequences were subcloned into the *Sac*I and *Xho*I restriction sites of the GP-miRGLO vector (GenePharma, Shanghai, China).

PC-12 cells were cultured in high glucose Dulbecco’s modified Eagle’s medium (Gibco) containing 5% FBS (Gibco) and 1% antibiotics (Gibco). The cells were incubated at 37 °C with 5% CO_2_. PC-12 cells with a confluency of 60–70% were transfected using luciferase reporter plasmids. After 24 h of culture, the cells were transfected using 0.5 µg of the GP-miRGLO plasmid (GenePharma, Shanghai, China) mixed with 1, 10, or 100 pM miR-182 agomir/antagomir using Lipofectamine 2000 (Invitrogen). The transfection was conducted in an antibody- and serum-free medium, according to the manufacturer’s instructions. After 6 hours, the medium was replaced with high glucose medium containing 5% FBS and 1% antibiotics. After an additional 30 h of culture, the transfected cells were lysed using 1× passive lysis buffer, and 20 µL of the supernatant was assayed for luciferase activity using the Dual-Luciferase Reporter Assay System (Promega). The relative reporter activity was calculated by normalizing the activity of *firefly* to *renilla* luciferase. Three replicates were used for each group.

### qRT-PCR

For quantitative real-time PCR, total RNA was extracted from L4-6 DRGs using the Trizol method (Invitrogen) and reverse-transcribed using ThermoScript reverse transcriptase (Thermo Fisher Scientific). The procedure was carried out as described in published studies^53,54^. The template (2 µL) was amplified using real-time PCR with the primers listed in Table 1. GAPDH was used as an internal control for normalization. Triplicate samples of 20 µL were used. Reactions were performed using a 7500 Fast Real-Time PCR detection system (Applied Biosystems, USA). The ratios of ipsilateral-side levels to contralateral-side levels were calculated using the −ΔΔCt method (2^−ΔΔCt^). All data were normalized to the GAPDH mRNA or U6, as they has been demonstrated to be stable^42,49,55^.

**Table 1:** Primer sets used in qPCR for rat samples.

Gene name	Primer sequence
SCN9A	5′-TGG CGT CGT GTC GCT TGT-3′
	5′-TGG CCC TTT GCC TGA GAT-3
GAPDH	5′-TCG GTG TGA ACG GAT TTG GC-3′
	5′-CCT TCA GGT GAG CCC CAG C-3
U6	5′-GCT TCG GCA GCA CAT ATA CTA A-3′
	5′-CGA ATT TGC GTG TCA TCC TT-3′
miR-182	5′-ACC TGG ATT TGG CAA TGG TAG-3′
	5′-TAT GCT TGT TCT CGT CTC TGT GTC-3′

### *In situ* hybridization (ISH) of miR-182 and single- or double-labeled immunofluorescence

Rats were anesthetized using isoflurane and then perfused with 4% paraformaldehyde before the DRGs were collected for single- or double-labeled immunohistochemistry. L4 and L5 DRGs were removed, post-fixed, and gradient dehydrated before frozen sectioning to a 16 µm thickness.

Cellular localization of miR-182 was performed using the rat miR-182 ISH Assay Kit (Boster Bio-Tech, Wuhan, China). Briefly, ISH was performed in 16 µm cryosections from the DRGs. Sections were fixed in 4% paraformaldehyde/0.1 M phosphate buffered saline (dissolved in DEPC-treated ultrapure water) for 30 min then washed in DEPC-treated ultrapure water. After treatment with 30% H_2_O_2_ mixed with methanol (v/v = 1:50) for 30 min, sections were treated with pepsase and diluted with 3% citric acid for 120 s at room temperature. Prehybridization procedures were performed at 42 °C for 4 h. Then, hybridization was carried out overnight in hybridization buffer at 42 °C using a hybridization probe specific to miR-182. After washing, the sections were incubated in blocking solution at 37 °C for 30 min and in mouse-anti-DIG-biotin for 60 min. The sections were then washed again, and were incubated using a SABC-FITC reagent for 30 min. To test whether SCN9A co-locates with miR-182, the sections were incubated overnight at 4 °C with primary antibodies against Nav1.7 (rabbit, 1:200, Abcam) following ISH. On the following day, a Cy3-conjugated secondary antibody (1:200, Jackson ImmunoReserch, West Grove, PA) was added, and sections were incubated for 2 h at room temperature. The signal was detected using a confocal microscope (Olympus Fluoview FV1000, Japan).

To identify the cell types expressing Nav1.7, sections were blocked for 1 h at room temperature in 0.01 M phosphate-buffered saline, containing 10% goat serum as well as 0.3% Triton X-100. The sections were then incubated with primary antibodies over 1 or 2 nights at 4 °C. The antibodies included: anti-Nav1.7 (rabbit, 1:200, Abcam), anti-calcitonin gene-related peptide (CGRP) (rat, 1:200, SIGMA, C8198), anti-lectin from *Bandeirae simplicifolia* BS-I Isolectin B4FITC Conjugate (1:200, Sigma), or anti-Neurofilament 200 (NF200) (mouse, 1:200, Boster Bio-Tech, Wuhan, China). On the following day, the sections were then incubated with either a donkey anti-rabbit antibody conjugated to Cy3 (1:200, Jackson ImmunoResearch, West Grove, PA), a donkey anti-mouse antibody conjugated to Alexa Fluor 488 (1:200, Jackson ImmunoResearch), or a donkey anti- mouse antibody conjugated to Cy3 (1:200, Jackson ImmunoResearch) for 2 h at room temperature. All the immunofluorescence-labeled sections were examined using a laser scanning confocal microscope (Olympus Fluoview FV1000, Japan). Single- or double-labeled neurons were quantified using Image J Software^56^.

### Western blotting

Protein was isolated from harvested unilateral L4-6 rat DRGs. The DRGs were homogenized and the cells were ultrasonicated in chilled lysis buffer. After centrifugation (15 min at 1000 × g at 4 °C), the supernatant was collected to isolate the protein. The concentration of the protein in the samples was measured using the Bio-Rad protein assay (Bio-Rad). The samples were boiled at 99 °C for 5 min and loaded onto a 5% stacking/6% separating SDS-polyacrylamide gel. The proteins were then transferred onto polyvinylidene difluoride membranes. After the membranes were blocked using 5% FBS for 2 h at room temperature, the following primary antibodies were applied: anti-Nav1.7 (mouse, 1:1000, Abcam) and anti-β-actin (mouse, 1:1000, Zhongshan Jinqiao, China). The proteins were detected using ECL detection reagents (Alphalmager proteinsimple, San Jose, USA) and exposed using FluorChem E (Alphalmager proteinsimple, San Jose, USA). The immunoreactive density was analyzed using Alpha Viem SA. All cytosol protein bands were normalized to β-actin^57^.

### DRG neuron preparation for patch clamp recording

Before DRG neuron preparation, glass coverslips (10 mm round, Assistant, Germany) were first cleaned by 75% (vol/vol) ethanol and air dried in a sterile culture hood. Cleaned coverslips were coated with poly-D-lysine (0.1 mg/ml, 90 μl per coverslip, O/N) and laminin (20 μg/ml, 5 μl per coverslip). Coated coverslips were sterilized again with UV light for 10 min before plating into the sterile 60 mm central well dish.

Seven days after SNI surgery or three days after microRNA agomir/antagomir microinjection, L4 and L5 DGRs were used for whole-cell patch clamp recording. After animals were euthanized with isoflurane, fresh DRGs ipsilateral and contralateral to SNI surgery site were quickly isolated and placed into cold Neurobasal Medium. The excess nerves and blood vessels were dissected under a stereoscopic microscope (Nikon, Japan) using micro scissors and forceps. The isolated DRGs were dissociated with 1 mg/ml collagenase type I and 2 mg/ml dispase in Neurobasal Medium for 30–35 min. DRG cell suspension was added to the dishes with pre-coated coverslips and placed into the incubator (37 °C, 5% CO2) for 2–4 h before recording.

### Whole-cell patch clamp recording

Whole-cell current-clamp recording was performed using pipettes (3–5 MΩ) fabricated from borosilicate capillary glass (P-97, Sutter Instruments, CA). Cell cultures were plated into a thermostatic chamber (Warner, USA) mounted on an inverted microscope (Nikon, Japan). The extracellular solution contained (in mM): 145 NaCl, 3 KCl, 2 MgCl_2_, 2 CaCl_2_, 10 HEPES, and 10 glucose (pH 7.4 with NaOH). The intracellular pipette solution contained (in mM): 135 KCl, 2 MgCl_2_, 2 Na_2_-ATP, 10 glucose, 10 HEPES, and 10 EGTA, with pH adjusted to 7.2 by KOH and osmolarity adjusted to 300 mOsm with sucrose. Signals were low-pass filtered at 2.9 kHz (EPC 10, HEKA, Germany) and digitized at 10 kHz using Patchmaster (HEKA, Germany) software. No holding current was injected into neurons. The bridge was 100% balanced. Action potentials (APs) were generated by injection of a series of current pulses (100 to 1500 pA in steps of 100 pA, 200 ms). The baseline potential had been recorded for 10 ms before the stimulus pulses were injected into the neurons. We defined the resting membrane potential (RMP) as the mean value of the 10 ms pre-stimulus potential in the first trial and the AP rheobase as the minimum current required to evoke the first AP.

### Statistical analysis

All of the results are presented as mean ± standard error of the mean. GraphPad Prism v5.0 was used for statistical analyses. The data were analyzed statistically using a two-tailed, paired Student’s *t*-test and one or two-way ANOVA. When the ANOVA showed a significant difference, pairwise comparisons between means were tested using the *post hoc* Tukey method. Significance is set at *p* < 0.05.

## Electronic supplementary material

Supplementary Figures

## Data Availability

The datasets generated during and/or analysed during the current study are available from the corresponding author on reasonable request.
