# Supplementary material for: MicroRNA-182 Alleviates Neuropathic Pain by Regulating Nav1.7 Following Spared Nerve Injury in Rats
Source: Sci Rep. 2018 Nov 13;8:16750. doi: 10.1038/s41598-018-34755-3 (PMC6233159; doi:10.1038/s41598-018-34755-3)
Supplement: Supplementary file 1 — Supplementary Figures [file 41598_2018_34755_MOESM1_ESM.pdf]

Supplementary Information Titles

Journal: Scientific reports

Article Title: MicroRNA-182 Alleviates Neuropathic Pain by Regulating Nav1.7 Following Spared Nerve Injury in Rats

Authors: Weihua Cai, Qingzan Zhao, Jinping Shao, Jingjing Zhang, Lei Li, Xiuhua Ren, Songxue Su, Qian Bai, Ming Li, Xuemei Chen, Jian Wang, Jing Cao, Weidong Zang

Corresponding Author: Weidong Zang, Jing Cao

Supplementary Item & Number:

1. **Supplementary figure 1** Full western blot images.
2. **Supplementary figure 2** Uptake of chemical modified small RNA sequence (miR-137 negative control) in DRG cells.

Supplementary figure 1

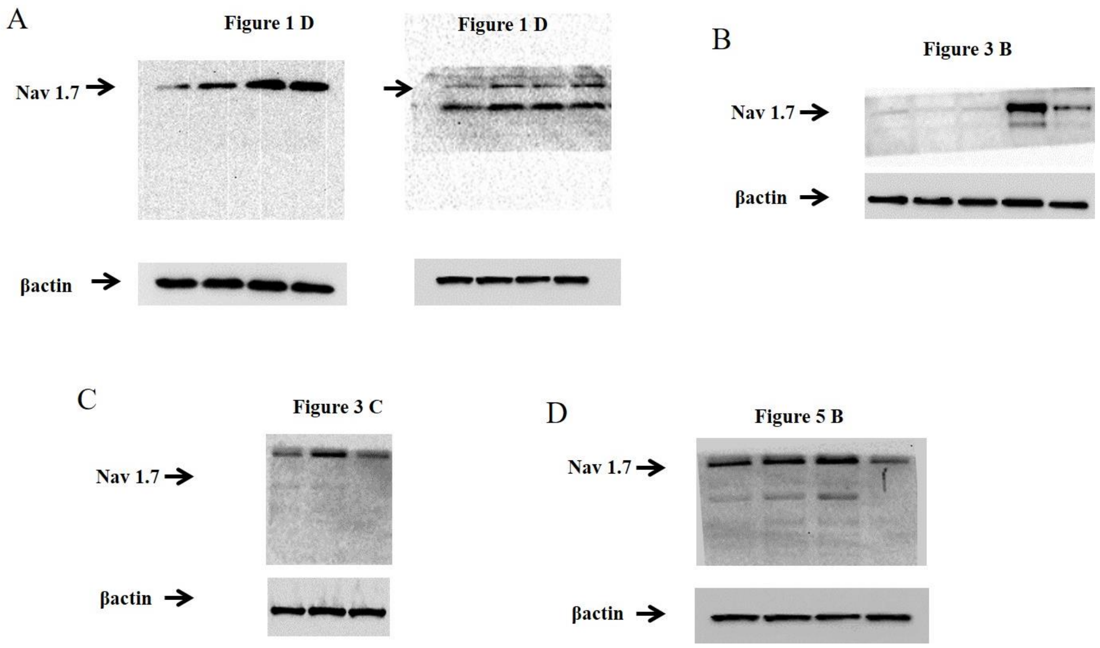

Supplementary figure 1 The original image of western blot results. (A-D) Original images for the results presented in Figure 1D (A), Figure 3B (B), Figure 3C (C) and Figure 5B (D).

Supplementary figure 2

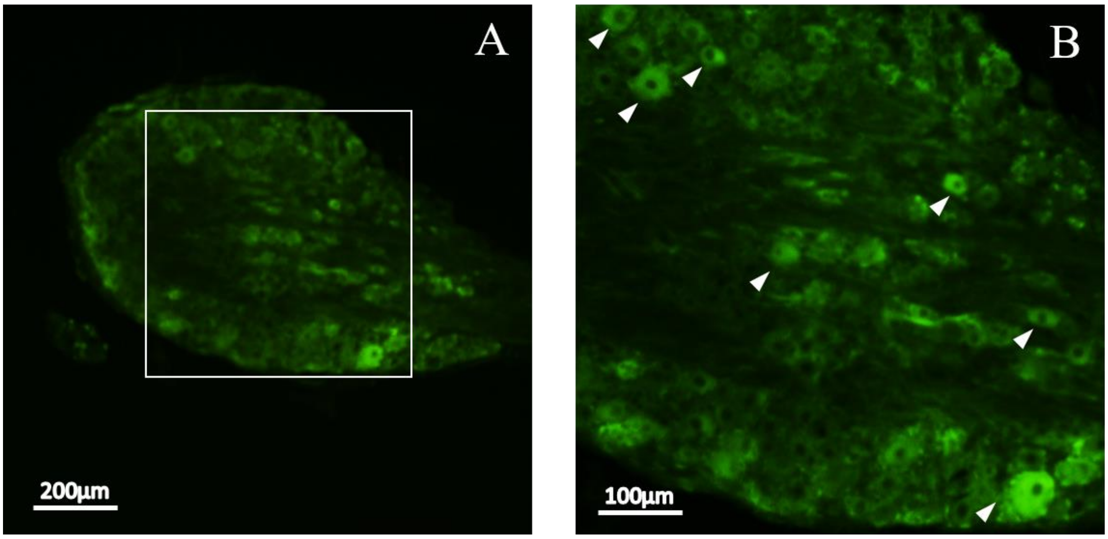

Supplementary figure 2 Uptake of chemical modified exogenous small RNA sequence (miR-137 negative control) in DRG cells. (A) Representative slice image. (B) The high magnification of area framed in A. White triangle: positive neurons.
